# Prediction model of delayed graft function based on clinical characteristics combined with serum IL-2 levels

**DOI:** 10.1186/s12882-022-02908-2

**Published:** 2022-08-15

**Authors:** Shitao Zhao, Yuan Liu, Chen Zhou, Zide Chen, Zeyu Cai, JiaLiang Han, Jiansheng Xiao, Qi Xiao

**Affiliations:** Department of Transplantation, The First Affiliated Hospital of Nanchang University, 17 Yongwai Zhengjie Street, Nanchang, 330006 Jiangxi China

**Keywords:** Kidney transplantation, Delayed graft function, Interleukin-2, Cold ischemia time, Diabetes, Creatinine

## Abstract

**Background:**

Kidney transplantation is an effective treatment for end-stage renal disease (ESRD). Delayed graft function (DGF) is a common complication after kidney transplantation and exerts substantial effects on graft function and long-term graft survival. Therefore, the construction of an effective model to predict the occurrence of DGF is particularly important.

**Methods:**

Seventy-one patients receiving their first kidney transplant at the First Affiliated Hospital of Nanchang University from October 2020 to October 2021 were enrolled in the discovery cohort. Based on clinical characteristics and serum markers, a logistic regression model was used to simulate the risk of DGF in the discovery cohort. The DGF prediction model was named the prediction system and was composed of risk factors related to DGF. Thirty-two patients receiving a kidney transplant at the First Affiliated Hospital of Nanchang University from October 2021 to February 2022 were enrolled in the validation cohort. The validation cohort was used to verify the accuracy and reliability of the prediction model.

**Results:**

Cold ischemia time (CIT), donor history of diabetes mellitus, donor interleukin-2 (IL-2) level and donor terminal creatinine level constitute the prediction system. In the validation test, the area under the receiver operating characteristic curve (AUC) was 0.867 for the prediction system, and good calibration of the model was confirmed in the validation cohort.

**Conclusions:**

This study constructed a reliable and highly accurate prediction model that provides a practical tool for predicting DGF. Additionally, IL-2 participates in the kidney injury process and may be a potential marker of kidney injury.

## Introduction

Kidney transplantation is an effective treatment for end-stage renal disease. However, the demand and supply disparity among patients waiting for kidney transplants and the availability of donor organs is widening. Expanded criteria donors (ECDs) are increasingly used for transplantation to alleviate the shortage of organs. However, the use of these donors has resulted in a significant increase in postoperative complications (e.g., delayed graft function (DGF)) [1]. Delayed graft function (DGF) is a common complication occurring after kidney transplantation and is associated with postoperative mortality, length of stay and long-term graft survival, among other factors [2, 3]. The definition of DGF is not completely unified at the present stage. The most widely accepted definition of DGF is a need for dialysis within one week after transplantation [4].

In recent years, transplant centers around the world have begun to build models to predict DGF by collecting preoperative information. Irish et al. [5] first published the DGF prediction model in 2003. Chapal et al. [6] included induction therapy characteristics in the DGF scoring system to improve the predictive power and increased the area under the receiver operating characteristic curve (AUC) to 0.73. However, the DGF prediction model constructed by Zaza et al. [7] only included recipient characteristics without donor characteristics, and the prediction efficiency was lower than that of the previous prediction models (AUC = 0.63). The Kidney Donor Risk Index (KDRI) scoring system [8] has been used for the preoperative prediction of DGF due to its correlation with the risk of DGF. However, the AUC of the KDRI is 0.67. The KDRI plays an important role in donor kidney allocation, which determines whether to accept kidneys from deceased donors, and it does not take into account a number of factors that cause DGF, such as cold ischemia time (CIT), the general condition of the recipient, immune status and other factors.

Clinical characteristics and preoperative kidney injury markers are also important for predicting the occurrence of DGF. Most studies have shown that acute kidney injury (AKI) caused by ischemia–reperfusion injury (IRI) is the main factor contributing to DGF because the production of cytotoxic mediators and activation of innate and adaptive immune responses after reperfusion all cause damage and necrosis of renal tubular cells [9–11]. Interleukins (ILs) play an important role in the immune system and are involved in the process of kidney injury, but they have not been used to assess prognosis after kidney transplantation.

Here, we discuss the involvement of serum markers in renal function impairment, construct a novel, reliable and highly accurate prediction model combine with serum markers, aiming to provide new insights for the DGF prediction after kidney transplantation.

## Materials and methods

### Study design

Sample and data collection were approved by the First Affiliated Hospital of Nanchang University ethical committees and were performed according to the Declaration of Helsinki. This study retrospectively collected the clinical characteristics of 39 donors and 77 recipients who underwent transplant surgery at the First Affiliated Hospital of Nanchang University from October 2020 to October 2021. The following classes of renal allograft donor organs or recipients were excluded from the study: (1) they had undergone retransplantation or were transplanted with organs other than the kidneys; (2) had a positive crossmatch or positive panel-reactive antibody (PRA > 10%); (3) had abnormal hepatic function; (4) patients who had autoimmune disease or received immunosuppressive therapy before the transplant procedure; (5) living related donor; (6) patients who displayed less than seven days of patient-and-graft survival (the diagnosis of DGF takes 1 week); and (7) patients who underwent a double-kidney transplant. Five recipients were excluded due to retransplantation, one recipient was excluded due to double kidney transplants. Finally, 71 recipients were included in the discovery cohort.

**Fig. 1:**
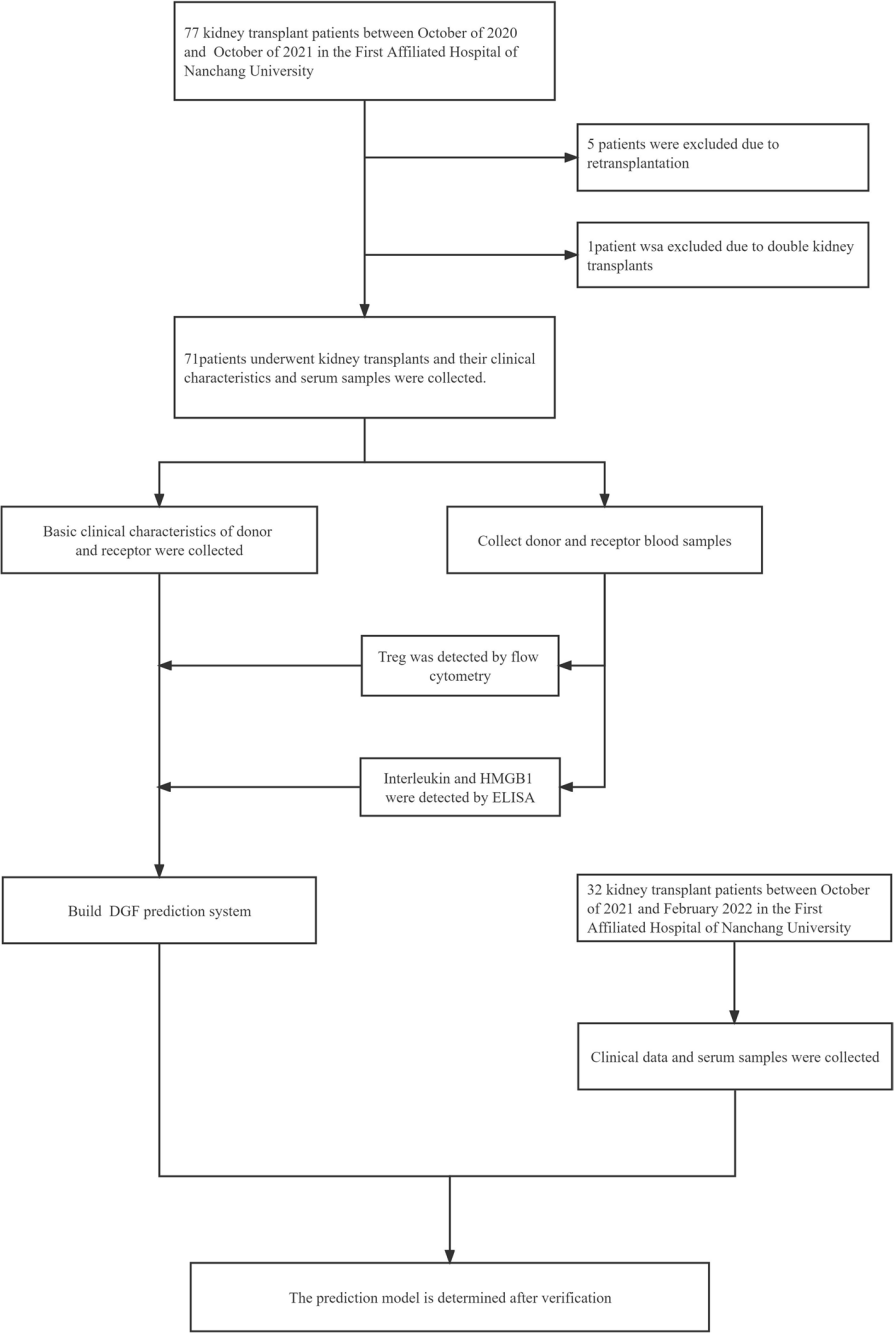
Flow chart showing the development of the prediction model

In addition, we collected information on 17 donors and 32 recipients who underwent transplant surgery at the First Affiliated Hospital of Nanchang University from October 2021 to February 2022 as a validation cohort to validate the predictive value of our model (Fig. 1).

### Sample collection and the experimental procedure

- **(1) Sample collection: **① Donor blood sample collection: When donor death was declared, donor peripheral blood samples (10 ml) were collected into a tube containing EDTA. ② Recipient blood sample collection: Recipient peripheral blood samples were collected into tubes (10 ml) containing EDTA before the induction of immunosuppression.

- **(2) Flow cytometry experimental procedure: **The frequency of Tregs was measured using flow cytometry. The main experimental equipment includes: CTYOMICS FC 500 flow cytometer, lysis solution (Beckman Coulter, Inc., CA, USA); CD4-FITC, CD25-PC5, and CD127-PE antibodies (BD Pharmingen, San Diego, CA, USA); and phosphate-buffered saline (Bwsm, Inc., Beijing, China).

- **(3) ELISA experimental procedure:** Plasma levels of interleukin-2 (IL-2), interleukin-4 (IL-4), interleukin-6 (IL-6), interleukin-10 (IL-10), cytotoxic T-lymphocyte-associated protein-4/cluster of differentiation 152 (CTLA-4/CD152), interleukin-35 (IL-35), and high mobility group Box 1 protein (HMGB1) were measured in the groups using commercially available enzyme-linked immunosorbent assay (ELISA) kits according to the manufacturer’s instructions. The main experimental equipment included IL-2, IL-4, IL-6, IL-10, CTLA-4/CD152, IL-35, and HMGB1 ELISA kits (ML Bio Inc., Shanghai, China).

### Study end points

DGF was defined as a need for dialysis in the first week after transplantation. Dialysis with heart failure was excluded. Patients who displayed less than seven days of patient-and-graft survival were excluded.

### Statistical analyses

Continuous variables are presented as the means ± standard deviations (SD) or medians (interquartile ranges [IQRs]), and categorical data are presented as percentages. Differences in continuous variables were analyzed using Student’s t tests or the Mann–Whitney U test. Categorical data were evaluated using the chi-square or Fisher exact test, as appropriate.

- **(1) Construction of the prediction model: **This analysis was performed based on the discovery cohort (n=71) using a multivariate logistic regression analysis. The independent predictors of DGF were identified if their P value was less than 0.05. The regression coefficients of the predictive variables in the logistic regression model were used to establish the nomogram to predict DGF.

- **(2) Validation of the prediction model: **Model efficacy was verified by analyzing the data from the discovery cohort (internal validation) and the validation cohort (external validation). ① Internal validation: The predictive ability of the prediction model was assessed using a c‑statistic of the receiver operating characteristic curve (ROC), and the 95% Confidence Interval (CI) of the areas under the ROC curves (AUCs) were nonparametrically obtained by bootstrap resampling (1000 bootstraps). The model prediction threshold was compared by performing decision curve analysis (DCA). By drawing a calibration diagram (1000 bootstraps), the performance characteristics of the model are shown graphically. We calculated the Brier score and R-squared values of the model. ② External validation: This analysis was repeated using data from the validation cohort. The validation cohort was divided into ten groups, and the DGF prediction model calibration was evaluated using the Hosmer‑Lemeshow statistic.

The statistical analyses were performed using SPSS software version 24.0 (SPSS Inc., Chicago, IL, USA), MedCalc statistical software version 19.6 (MedCalc Software Ltd., Ostend, Belgium) and R version 4.12 (R Project for Statistical Computing, Vienna, Austria).

## Results

### Donor, recipient, and transplant characteristics

A total of 103 patients were enrolled in the discovery cohort (*n* = 71) and validation cohort (*n* = 32). Table 1 shows no significant differences in the demographic and clinical characteristics between the discovery cohort and validation cohort. Table 2 lists the differences in recipient and donor baseline characteristics between the DGF group and the immediate graft function (IGF) group. In the discovery cohort, the donor terminal creatinine level in the IGF group (0.80 (0.67–1.08)) was significantly lower than that in the DGF group (1.52 (0.87–4.24)) (*P* = 0.001). The proportion of patients with a donor history of hypertension in the IGF group (17 (35.4%)) was lower than that in the DGF group (15 (65.2%)) (*P* = 0.018). The proportion of donors with a history of diabetes mellitus in the IGF group (6 (12.5%)) was also lower than that in the DGF group (10 (43.5%)) (*P* = 0.003). The CIT in the IGF group (8.36 ± 2.27) was significantly shorter than that in the DGF group (10.61 ± 2.82) (*P* = 0.001). In contrast, the proportion of a recipient history of hypertension in the IGF group (44 (91.7%)) was higher than that in the DGF group (17 (73.9%)) (*P* = 0.044). Table 3 lists the differences in recipient and donor serum markers and Treg levels between the DGF group and the IGF group. In the discovery cohort, the donor IL-2 level in the IGF group (74.23 ± 23.96) was lower than that in the DGF group (96.42 ± 26.46) (*P* = 0.001). No significant differences in the levels of other serum markers or Tregs were observed between the groups.

**Table 1:** Recipient and donor characteristics at time of transplantation in the discovery cohort (*n* = 77) and validation cohort (*n* = 32)

*variable*	*Discovery cohort (n* = *71)*	*Validation cohort (n* = *32)*	*P*
***Recipient characteristics***			
Age, yr	37.97 ± 11.49	37.28 ± 13.50	0.790
Gender			0.333
Male	47(66.2%)	18(56.3%)	
Female	24(33.8%)	14(43.8%)	
BMI, kg/m^2^	21.44(18.67–24.36)	20.10(18.11–23.53)	0.259
Time on dialysis, m	24(15–60)	24(12–59.25)	0.338
Dialysis, HD	51(71.8%)	19(59.4%)	0.403
Immunosuppression regimen			0.267
ATG,Tac, MMF, steroid	15(21.1%)	10(31.3%)	
Basiliximab,Tac, MMF,steroid	61(85.9%)	24(75.0%)	
Pre-Tx Creatinine,mg/dL	12.05 ± 3.68	11.03 ± 3.43	0.189
history of hypertension	61(85.9%)	22(78.6%)	0.177
history of diabetes mellitus	3(4.2%)	0(0%)	0.238
***Donor characteristics***			
Age, (yr)	44(17–48)	29.5(15–51.25)	0.471
Gender			0.545
Male	57(80.3%)	24(75.0%)	
Female	14(19.7%)	8(25.0%)	
BMI, kg/m^2^	22.86(18.81–24.22)	23.75(18.11–24.80)	0.526
Terminal Creatinine, mg/dL	0.88(0.67–1.52)	0.72(0.59–1.46)	0.279
The donor type			0.155
DCD	13(18.3%)	4(12.5%)	
DBD	52(73.2%)	28(87.5%)	
DBCD	6(8.5%)	0(0%)	
Donor history of hypertension	32(45.1%)	10(31.3%)	0.187
Donor history of diabetes mellitus	16(22.5%)	3(9.4%)	0.111
*Transplant characteristics*			
CIT,h	9(7–11)	9(7–9.375)	0.309
HLA mismatches	5(4–5)	4(4–5)	0.371

Continuous variables were expressed as the mean ± SD or IQR, categorical data were expressed as n%

*ATG* Anti-thymocyte globulin, *Tac* Tacrolimus, *MMF* Mycophenolate Mofetil, *Pre-Tx* Pre-transplant, *HD* Hemodialysis, *CIT* Cold ischemia time, *HLA* Human leukocyte antigen, *DCD* Donation after cardiac death, *DBD* Donation after brain death, *DBCD* Donation after brain death plus cardiac death, *BMI* Body mass index, *NA* Not available

**Table 2:** The recipient and donor characteristics of DGF and IGF in the Discovery cohort and the validation cohort

*variable*	*Discovery cohort (n* = *71)*		*Validation cohort (n* = *32)*			
	***DGF(n* = *23)***	***IGF(n* = *48)***	***p***	***DGF(n* = *12)***	***IGF(n* = *20)***	***P***
***Recipient characteristics***						
Age, yr	39.26 ± 10.767	37.35 ± 11.883	0.517	36.67 ± 13.30	37.65 ± 13.95	0.846
Gender			0.904			0.854
Male	15(65.2%)	32(66.7%)		7(58.3%)	11(55.0%)	
Female	8(34.8%)	16(33.3%)		5(41.7%)	9(45.0%)	
BMI, kg/m^2^	22.53 ± 5.01	21.49 ± 3.58	0.317	20.38 ± 4.23	20.98 ± 3.20	0.654
Time on dialysis, m	36(24–84)	24(12.5–49.5)	0.110	24(13.75–29.75)	22(12–60)	0.876
Dialysis,HD	16(69.6%)	35(72.9%)	0.506	9(75.0%)	10(50%)	0.289
Immunosuppression regimen			0.930			0.076
ATG,Tac, MMF, steroid	5(21.7%)	10(20.8%)		6(50%)	4(20.0%)	
Basiliximab,Tac, MMF,steroid	18(78.3%)	38(79.2%)		6(50%)	16(80.0%)	
Pre-Tx Creatinine, mg/dL	12.04 ± 3.53	12.05 ± 3.79	0.988	11.19 ± 3.51	10.93 ± 3.48	0.841
history of hypertension	17(73.9%)	44(91.7%)	**0.044**	8(66.7%)	16(80.0%)	0.399
history of diabetes mellitus	0(0%)	3(6.3%)	0.221	0(0%)	0(0%)	NA
***Donor characteristics***						
Age, yr	43(35–47)	46(17–49.75)	0.631	38.50 ± 18.76	27.70 ± 15.87	0.092
Gender			0.106			0.092
Male	21(91.3%)	36(75.0%)		11(91.7%)	13(65.0%)	
Female	2(8.7%)	12(25.0%)		1(8.3%)	7(35.0%)	
BMI, kg/m^2^	23.39(18.37–24.46)	22.49(19.42–24.22)	0.671	24.62(18.53–25.48)	21.35(18.03–24.46)	0.226
Terminal Creatinine, mg/dL	1.52(0.87–4.24)	0.80(0.67–1.08)	**0.001**	0.37(0.92–1.74)	0.67(0.56–0.80)	**0.003**
The donor type			0.490			0.581
DCD	6(26.1%)	7(14.6%)		2(16.7%)	2(10%)	
DBD	15(65.2%)	37(77.1%)		10(83.3%)	18(90%)	
DBCD	2(8.7%)	4(8.3%)		NA	NA	
Donor history of hypertension	15(65.2%)	17(35.4%)	**0.018**	4(33.3%)	2(10.0%)	0.102
Donor history of diabetes mellitus	10(43.5%)	6(12.5%)	**0.003**	3(25.0%)	0(0%)	**0.019**
***Transplant characteristics***						
CIT,h	10.61 ± 2.82	8.36 ± 2.27	**0.001**	9.5 ± 1.23	7.95 ± 1.34	**0.006**
HLA mismatches	5(4–6)	5(4–5)	0.663	4(4–5.75)	4.5(4–5)	0.637

Continuous variables were expressed as the mean ± SD or IQR, categorical data were expressed as n%

*ATG* Anti-thymocyte globulin, *Tac* Tacrolimus, *MMF* Mycophenolate Mofetil, *Pre-Tx* Pre-transplant, *HD* hemodialysis, *CIT* Cold ischemia time, *HLA* Human leukocyte antigen, *DCD* Donation after cardiac death, *DBD* Donation after brain death, *DBCD* Donation after brain death plus cardiac death, *BMI* Body mass index; NA: Not available

**Table 3:** The Donor and recipient serum markers level of DGF and IGF in discovery and validation cohort

*Variables*	*Discovery cohort (n* = *71)*		*Validation cohort (n* = *32)*			
	***IGF(n* = *48)***	***DGF(n* = *23)***	***p***	***IGF(n* = *20)***	***DGF(n* = *12)***	***p***
***Donor***						
IL-2(pg/mL)	74.23 ± 23.96	96.42 ± 26.46	**0.001**	76.22(63.25–95.64)	97.70(89.56–113.83)	**0.003**
IL-4(pg/mL)	4.55 ± 1.87	3.96 ± 2.17	0.240			
IL-6(pg/mL)	6.04 ± 1.55	5.37 ± 2.12	0.138			
IL-10(pg/mL)	67.33 ± 31.53	73.59 ± 26.19	0.412			
CTLA-4;CD152(pg/mL)	137.91 ± 58.81	136 ± 71.36	0.909			
IL-35(pg/mL)	9.08(5.22–10.43)	9.67(5.79–10.78)	0.632			
HMGB1(pg/mL)	5643.15 ± 1493.09	5937.63 ± 1968.60	0.486			
***recipient***						
IL-2(pg/mL)	99.62 ± 23.45	94.87 ± 20.93	0.411			
IL-4(pg/mL)	5.95 ± 1.79	6.26 ± 1.19	0.454			
IL-6(pg/mL)	7.65 ± 1.50	7.39 ± 1.41	0.481			
IL-10(pg/mL)	92.76 ± 21.57	89.61 ± 20.24	0.559			
CTLA-4;CD152(pg/mL)	214.50 ± 54.42	213.83 ± 50.69	0.961			
IL-35(pg/mL)	11.01 ± 2.11	11.05 ± 2.86	0.944			
Treg (%)	5.10 ± 2.39%	5.34 ± 2.53%	0.700			

*CTA* Cytotoxic T-lymphocyte-associated protein, *CD* Cluster of differentiation, *IL* Interleukin, *HMGB1* High mobility group box 1 protein, *Treg* Regulatory T cells

### Univariate and multivariate analyses and construction of the DGF prediction model

**Fig. 2:**
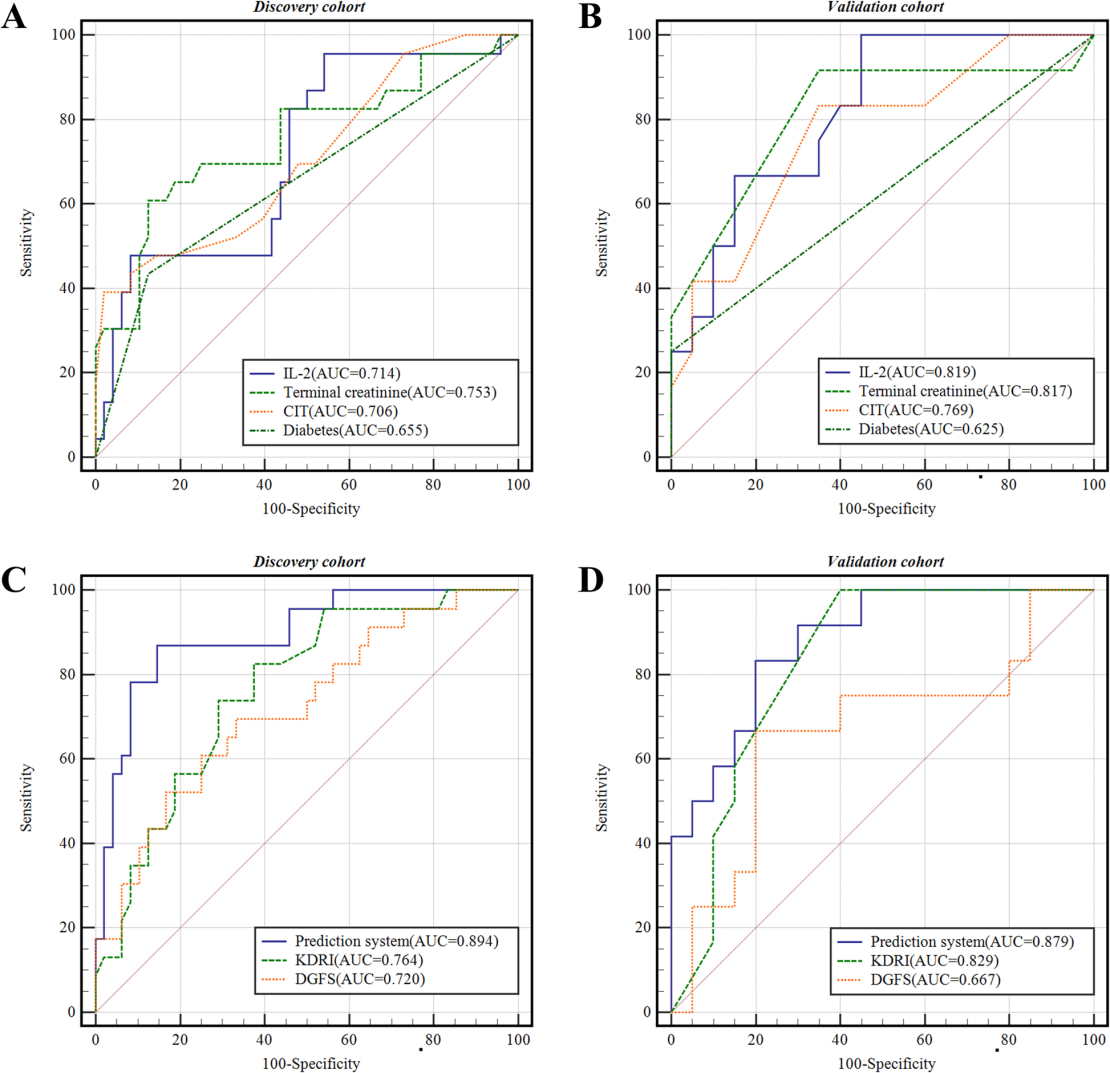
The relative efficiencies for predicting DGF using receiver operating characteristic curves (ROC). **A** ROC curves were constructed to evaluate the predictive power of independent risk factors in the discovery cohort. **B** ROC curves were constructed to evaluate the predictive power of independent risk factors in the validation cohort. **C** ROC curves were constructed to evaluate the predictive power of different prediction models in the discovery cohort. **D** ROC curves were constructed to evaluate the predictive power of different prediction models in the validation cohort

**Fig. 3:**
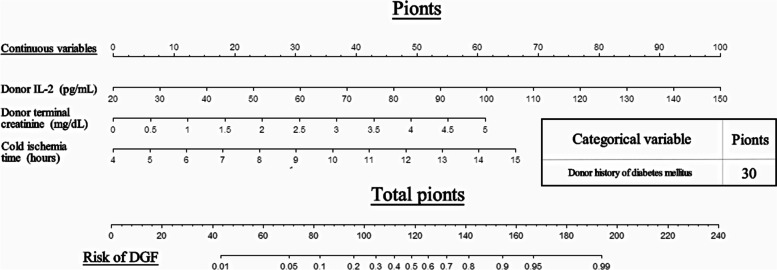
A nomogram predicting the risk of DGF in kidney transplant recipients

Tables 4 and 5 shows the results of univariate analyses and multivariate analyses of the discovery cohort. Univariate logistic analysis showed that donor terminal creatinine levels, donor history of hypertension, donor history of diabetes mellitus, CIT, and donor IL-2 levels were related to postoperative DGF. After adjusting for the effects of the factors listed above in the multivariate logistic regression analysis, CIT, donor history of diabetes mellitus, donor IL-2 levels and donor terminal creatinine levels were considered independent risk factors for DGF. The ROC curve was plotted to evaluate the prediction model and calculate the AUCs (Fig. 2(A)). The AUCs were 0.753 for donor terminal creatinine levels, 0.655 for a donor history of diabetes mellitus, 0.706 for the CIT, and 0.714 for donor IL-2 levels (Table 6). Finally, these four variables (donor terminal creatinine levels, donor history of diabetes mellitus, CIT, and donor IL-2 levels) constituted the nomogram model (named the prediction system) shown in Fig. 3 to predict DGF after kidney transplantation. The DGF prediction model was calculated using the formula (-9.6319 + 0.368 × CIT + 1.789 × donor history of diabetes mellitus + 0.047 × donor IL-2 level + 0.749 × donor terminal creatinine level).

**Table 4:** Results of the univariate logistic regression analysis

*Variables*	*Univariate analysis*		
	***OR***	***95%CI***	***P***
***Recipient characteristics***			
Age, yr	1.015	(0.971–1.061)	0.511
Gender	0.938	(0.329–2.671)	0.904
BMI, kg/m^2^	1.065	(0.942–1.203)	0.313
Time on dialysis, m	1.008	(0.995–1.021)	0.239
Dialysis,HD	1.295	(0.534–3.143)	0.568
Immunosuppression regimen	1.056	(0.314–3.544)	0.930
Pre-Tx Creatinine, mg/dL	1.000	(0.988–1.002)	0.987
history of hypertension	0.258	(0.065–1.027)	0.055
history of diabetes mellitus	NA	NA	NA
***Donor characteristics***			
Age, yr	1.004	(0.976–1.032)	0.787
Gender	3.500	(0.713–17.176)	0.123
BMI, kg/m^2^	1.022	(0.890–1.173)	0.760
Terminal Creatinine, mg/dL	2.279	(1.395–3.722)	**0.001**
The donor type			0.499
DCD	REF		
DBD	1.714	(0.228–12.890)	0.601
DBCD	0.811	(0.134–4.907)	0.819
Donor history of hypertension	3.419	(1.206–9.695)	**0.021**
Donor history of diabetes mellitus	5.385	(1.641–17.664)	**0.005**
***Transplant characteristics***			
CIT,h	1.438	(1.143–1.809)	**0.002**
HLA mismatches	1.000	(0.692–1.444)	0.998
***Donor serum markers***			
IL-2(pg/mL)	1.036	(1.013–1.059)	**0.002**
IL-4(pg/mL)	0.857	(0.663–1.107)	0.238
IL-6(pg/mL)	0.800	(0.595–1.076)	0.140
IL-10(pg/mL)	1.007	(0.990–1.025)	0.407
CTLA-4(pg/mL)	1.000	(0.992–1.008)	0.908
IL-35(pg/mL)	1.002	(0.834–1.205)	0.982
HMGB1(pg/mL)	1.000	(1.000–1.000)	0.481
***Recipient serum markers***			
IL-2(pg/mL)	0.990	(0.968–1.013)	0.990
IL-4(pg/mL)	1.128	(0.826–1.539)	0.449
IL-6(pg/mL)	0.881	(0.623–1.247)	0.476
IL-10(pg/mL)	0.993	(0.969–1.017)	0.553
CTLA-4(pg/mL)	1.000	(0.990–1.009)	0.960
IL-35(pg/mL)	1.008	(0.815–1.245)	0.943
Treg frequency(%)	1.042	(0.848–1.281)	0.695

*ATG* Anti-thymocyte globulin, *Tac* Tacrolimus, *MMF* Mycophenolate Mofetil, *Pre-Tx* Pre-transplant, *HD* Hemodialysis, *CIT* Cold ischemia time, *HLA* Human leukocyte antigen, *DCD*, Donation after cardiac death, *DBD* Donation after brain death, *DBCD* Donation after brain death plus cardiac death, *BMI* Body mass index, *CTA* cytotoxic T-lymphocyte-associated protein, *CD* Cluster of differentiation, *IL* Interleukin; *HMGB1* high mobility group box 1 protein, *Treg* Regulatory T cells; *NA* Not available

**Table 5:** Results of the multivariable logistic regression analysis

*Variables*	*Multivariate analysis*		
	***OR***	***95%CI***	***P***
***Donor characteristics***			
Terminal Creatinine, mg/dL	0.749	1.054–4.245	**0.035**
Donor history of diabetes mellitus	1.789	1.191–30.048	**0.030**
***Transplant characteristics***			
CIT,h	0.368	1.046–1.995	**0.026**
***Donor serum markers***			
IL-2(pg/mL)	0.047	1.017–1.080	**0.003**

*CIT* Cold ischemia time

**Table 6:** The predictive value of prognosis models

	**ROC Area (95% CI)**	**Cut-Off Point**	**Sensitivity (%)**	**Specificity (%)**
***Discovery cohort***				
Donor Terminal Creatinine(mg/dL)	0.753(0.622–0.883)	1.3178	60.9%	87.5%
Donor history of diabetes mellitus	0.655(0.510–0.799)	0.5	43.5%	87.5%
CIT(h)	0.706(0.573–0.839)	11.25	43.5%	91.7%
Donor IL-2(pg/mL)	0.714(0.585–0.843)	66.415	95.7%	45.8%
Prediction_system	**0.894(0.798–0.955)**	-0.896	86.96%	85.42%
KDRI	**0.764(0.649–0.857)**	1.13	82.61%	62.50%
DGFS	**0.720(0.601–0.820)**	0.2767	69.57%	66.67%
***Validation cohort***				
Donor Terminal Creatinine(mg/dL)	0.817(0.640–0.931)	0.676	91.7%	65%
Donor history of diabetes mellitus	0.625(0.437–0.789)	0	25%	100%
CIT(h)	0.769(0.586–0.899)	8	83.33%	65%
Donor IL-2(pg/mL)	0.819(0.643–0.932)	87.11	100%	55%
Prediction_system	**0.879(0.715–0.967)**	-0.984	0.8333	0.800
KDRI	**0.829(0.655–0.938)**	0.80	100%	60%
DGFS	**0.667(0.479–0.823)**	0.327	66.7%	80%

*KDRI* The Kidney Donor Risk Index, *DGFS*, Delayed graft function score, *CIT* Cold ischemia time

### Evaluating the predictive capacity of the DGF prediction model

**Fig. 4:**
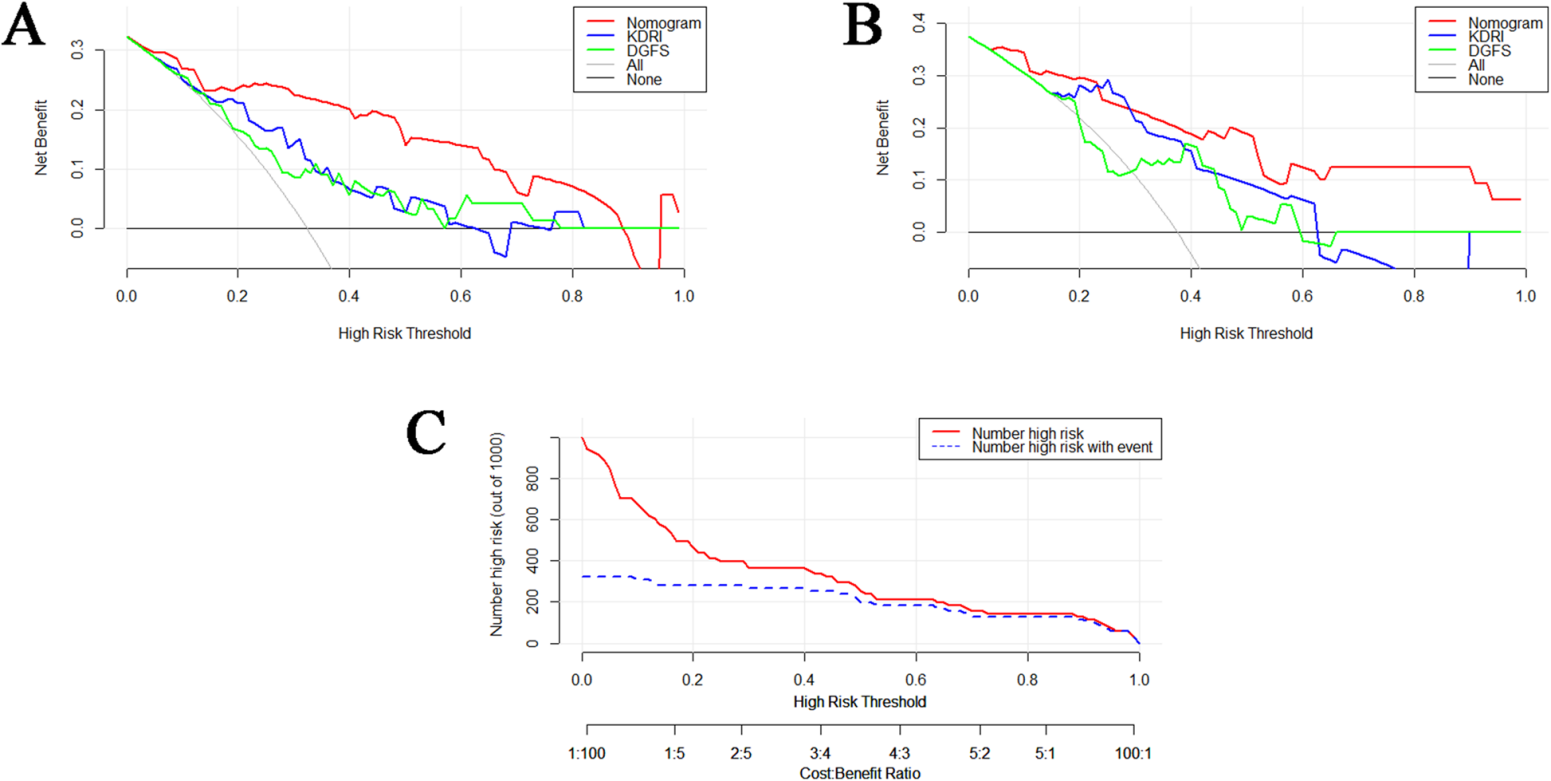
Evaluation of the predictive validity of the model using DCA and CIC. **A** and **B** The DCA curves for the discovery and validation cohorts, respectively. **C** CIC of the prediction model

We used AUC, decision curve analysis (DCA), and clinical impact curve (CIC) to evaluate the predictive power of the prediction system. The relative efficiencies of the KDRI, delayed graft function score (DGFS) and the prediction system for predicting DGF are shown in Figs. 2 and 4.

#### Internal validation

**Fig. 5:**
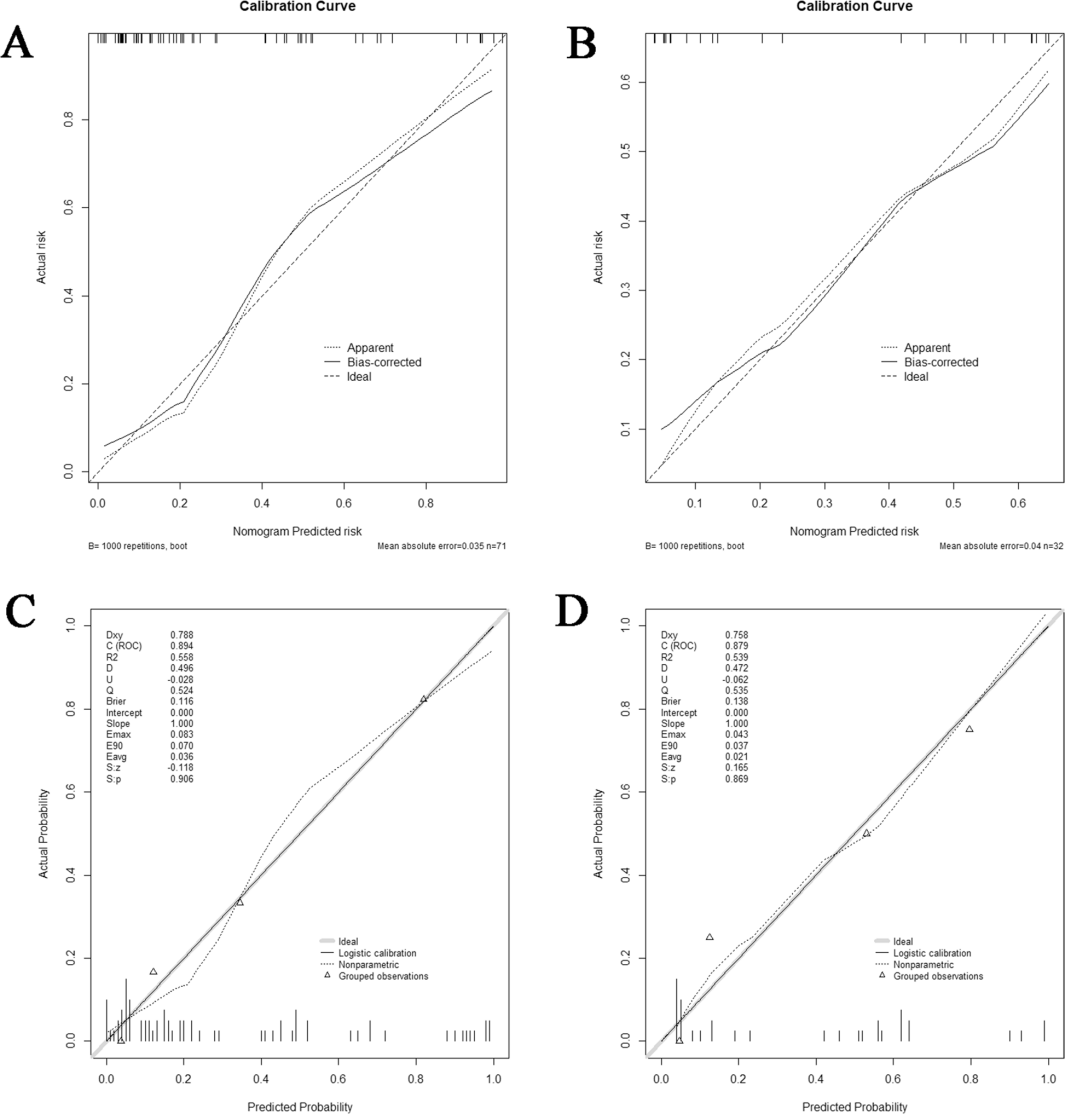
The calibration curve of the prediction system. **A** Calibration curve for internal validation using data from the discovery cohort. **B** The calibration curve for external validation using data from the validation cohort. **C** The calibration curve of the internally validated nomogram for the discovery cohort using the Brier score and R-squared values. **D** The calibration curve of the externally validated nomogram for the discovery cohort using the Brier score and R-squared values

In the discovery cohort, the AUCs were 0.894 for the prediction system, 0.764 for the KDRI and 0.720 for the DGFS (Fig. 2C). As shown in Fig. 4A, when the threshold value (P_t_) is 0–0.88, the positive probability of DGF is higher with our prediction model, while the threshold value of KDRI(P_t_ = 0–0.75) and DGFS (P_t_ = 0–0.77) are narrower. Figure 4C shows that the positive rate predicted by the prediction system is basically the same as the actual positive rate. Figure 5A shows the calibration plot (1000 bootstraps) of the prediction system for the discovery cohort. The bias-corrected solid line represents the prediction system performance, which is close to the ideal. Figure 5C shows the Brier score (0.116) and R-squared value (55.8%) of the prediction system for the discovery cohort (a lower Brier score reflects a more accurate prediction of DGF, and < 0.25 indicates good calibration). All results indicate that the prediction system has good prediction efficiency.

#### External validation

The validation cohort consisted of 32 kidney transplant recipients for external validation. In the validation cohort, CIT was significantly shorter in the IGF group (7.95 ± 1.34) than in the DGF group (9.5 ± 1.23). The proportion of donors with a history of diabetes mellitus in the IGF group was lower than that in the DGF group. The donor IL-2 level in the IGF group (76.22 (63.25–95.64)) was lower than that in the DGF group (97.70 (89.56–113.83)). The donor terminal creatinine level was significantly different between the DGF (1.37 (0.92–1.74)) group and IGF (0.67 (0.56–0.8)) group. Figure 2(B) shows the predictive efficiency of these four variables in the validation cohort. The AUCs were 0.817 for donor terminal creatinine levels, 0.625 for a donor history of diabetes mellitus, 0.769 for the CIT, and 0.819 for donor IL-2 levels (Table 6).

**Fig. 6:**
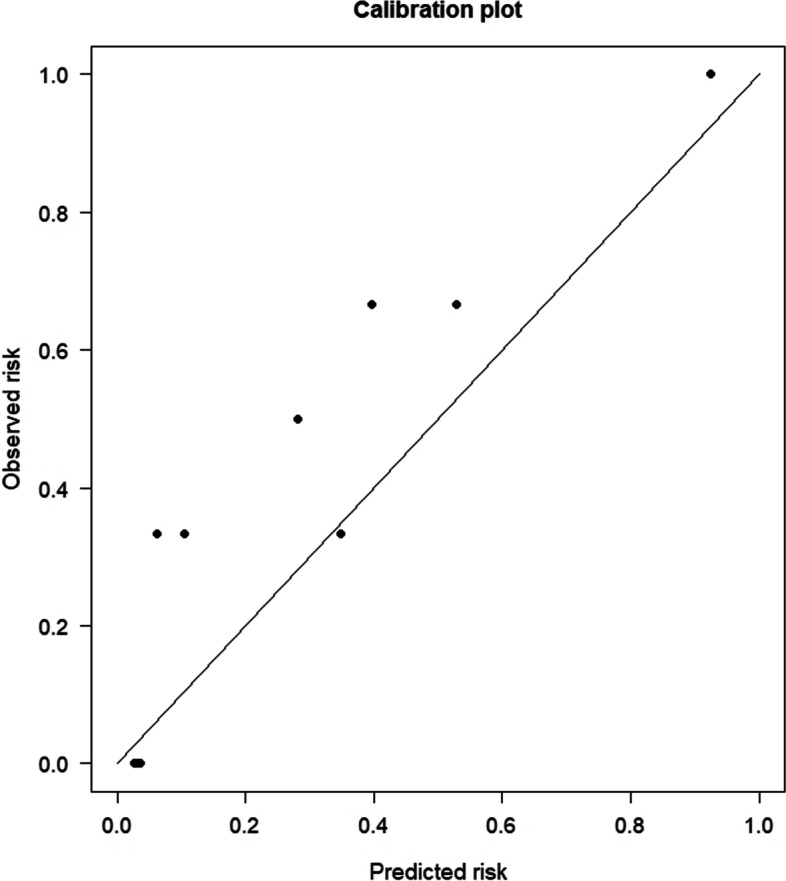
The calibration of the prediction system was evaluated using the Hosmer–Lemeshow test

In the validation cohort, the AUC calculated using the prediction system was 0.879, the KDRI was 0.829, and the DGFS was 0.667 (Fig. 2D). In the validation cohort, our prediction model still maintained a high accuracy. As shown in Fig. 4B, our prediction model had a broad threshold, while the KDRI (P_t_ = 0.16–0.62) and DGFS (P_t_ = 0.3–0.59) thresholds were still low. Our prediction model was better than KDRI in terms of decision-making ability. Figure 5B shows the calibration plot of the prediction system for the validation cohort. The bias-corrected solid line was also close to the ideal. Figure 5D shows the Brier score (0.138) and R-squared value (53.9%) of the prediction system for the validation cohort. The prediction system calibration was evaluated again using the Hosmer–Lemeshow statistic. Figure 6 illustrates the good calibration of the model for the validation cohort; the points in the image are close to the midline. The predicted and observed risks were similar (*P* = 0.4117, > 0.05 indicates good calibration).

## Discussion

DGF is one of the common postoperative complications after kidney transplantation and exerts a substantial effect on graft function and long-term graft survival [2, 3]. The occurrence of DGF was significantly reduced by changing the organ preservation strategy [12]. Therefore, building a reliable and accurate prediction model is particularly important to address the problems encountered by clinical decision-makers.

In our study, IL-2 was innovatively added to the prediction model. In 1983, IL-2 was discovered as an autocrine growth factor for cultured T cells [13]. IL-2 is involved in the proliferation of T cells and natural killer (NK) cells in the immune system, and may indirectly lead to kidney damage. First, weakening the ability of IL-2 to stimulate T cell proliferation has been shown to reduce ischemia–reperfusion injury (IRI) [14–17]. A study found that in the process of renal ischemia and reperfusion, IL-2 promotes NK cell proliferation, and NK cells directly kill renal tubular epithelial cells (TECs) [18]. Because renal parenchymal cells are mostly TECs, thus excessive apoptosis of TECs may lead to impaired renal function, indicating a potential link between NK cells and kidney injury. In addition to causing kidney damage by inducing the proliferation of immune cells, IL-2 has been reported to regulate TECs directly, leading to kidney injury. According to a previous study, IL-2 regulates cellular FLICE-inhibitory protein (C-FLIP) in TECs to increase the expression of endogenous caspase-8 in TECs, leading to TEC apoptosis and impaired renal function [19]. IL-2 is also widely used to treat immunodeficiency diseases, but the complications of impaired renal function often occur during treatment [20]. In our study, the level of IL-2 in the donor serum was positively correlated with DGF. This result may be related to the involvement of IL-2 in renal injury.

Creatinine is the product of phosphocreatine decomposition in muscle. It is produced at a fairly constant rate in the body, filtered freely through the glomerular membrane, and discharged almost completely through the kidney [21]. It is a clinically recognized sign of renal function [22]. The use of donor serum creatinine levels to evaluate renal function is a simple, efficient and cost-effective method. However, serum creatinine levels are influenced by other factors, such as diet, age, medications, and other factors, and the effects of other variables must be considered when evaluating kidney functions [23]. In our study, the donor serum creatinine level was included in the prediction model, and other risk factors were combined to improve the accuracy of the prediction. Similarly, the donor serum creatinine level was included in the KDRI model [8], and the model reported by Irish et al. [5].

Elevated blood glucose levels potentially lead to renal microvascular formation, glomerular basement membrane thickening, mesangial dilation, nodular glomerulosclerosis and tubulointerstitial fibrosis, which are the main pathological changes associated with diabetic nephropathy, and diabetes is the most common cause of ESRD [24]. Studies have shown that diabetes is a risk factor for AKI, and kidney damage directly increases the risk of DGF after transplantation [25]. Multiple studies have identified links between diabetes and inflammatory markers, such as tumor necrosis factor-α, interleukin-6, and C-reactive protein [26–30]. These inflammatory markers are independent risk factors for DGF and are associated with kidney injury [31–33]. An animal study found that elevated glucose levels in diabetic mice cause persistent kidney damage during warm ischemia–reperfusion, and diabetic mice are more prone to DGF than nondiabetic mice [34]. In our study, the number of donors with a history of diabetes in the DGF group was greater than that in the IGF group. In the multivariate analysis, a donor history of diabetes was an independent risk factor for DGF.

CIT is an important factor affecting the recovery of renal graft function, and a CIT reaching 12 h will increase the probability of primary graft failure and vascular complications [35]. Most studies have found that CIT is a susceptibility factor for renal injury and is closely related to the occurrence of DGF [36–38]. Thus, a shorter CIT may reduce the incidence of DGF and improve graft outcomes. In this study, the mean CIT (10.61 ± 2.82) in the DGF group was approximately 12 h, which represented a significant difference compared with the mean CIT (8.36 ± 2.27) of the IGF group. In the multivariate analysis, CIT was an independent risk factor for DGF. During the management of high-risk organs, CIT should be minimized as much as possible, and the occurrence of DGF might be reduced by controlling CIT within 12 h.

Jesper et al. [39] used four predictive models reported by Irish et al. [40], Jeldres et al. [41], Chapal et al. [6], and Zaza et al. [7] to predict the occurrence of DGF, and the range of the C-statistic was 0.567–0.761, indicating that different parameter combinations might improve the predictive value of DGF in different populations. In 2009, the KDRI was proposed by Rao et al. for graft assessment and decision-making using donor factors. Although KDRI reflects graft and patient survival, its AUC for predicting the occurrence of DGF in this study was 0.764, which is limited because it only included a few clinical factors affecting prognosis. Chapal et al. [6] published a simple DGFS based on a multicenter and prospective cohort in France. It has a good predictive capacity (AUC at 0.73). By calculating the five explanatory variables of the model, a patient with DGFS < -0.50 was predicted to have no DGF, and the accuracy rate was 88%. In contrast, among patients with a DGFS > 1.2, half will experience a DGF. Therefore, this model is widely used in Europe. In the present study, we found that donor terminal creatinine levels, a donor history of diabetes mellitus, CIT and donor IL-2 levels were related to the occurrence of DGF. The AUC, a measure of the diagnostic accuracy of risk factors for DGF, was 0.753, 0.655, 0.706 and 0.714 for donor terminal creatinine levels, a donor history of diabetes mellitus, CIT and donor IL-2 levels, respectively.). We constructed a nomogram using these four independent risk factors to improve the accuracy of the prediction. The predictive ability of the model (AUC = 0.894) was better than that of the KDRI and DGFS.

This study has several limitations. First, this study was performed at a single-center with a small sample size, and data from other centers have not been tested. Therefore, we hope that these findings will encourage external validation of the proposed model using data from more centers. In addition, this sample only covers Asian races, which has certain limitations in the process of promotion and application. Laboratory data are not dynamically observed and may cause bias. Finally, the scarce possibility of widespread use of the model due to the challenge represented by IL-2 determination in real life scenarios.

In summary, we constructed a DGF prediction model with high accuracy by including four factors (donor terminal creatinine levels, donor history of diabetes mellitus, CIT, and donor IL-2 levels) to provide clinicians with a useful tool that helps clinical decision-makers intervene more quickly and reduce the occurrence of DGF. IL-2 also participates in the kidney injury process and may be a potential marker of kidney injury.

## Data Availability

The datasets used and analyzed during the current study available from the corresponding author on reasonable request.
